# Effects of a Tailored Home-Based Exercise Program, “KidMove”, on Children with Cystic Fibrosis: A Quasi-Experimental Study

**DOI:** 10.3390/healthcare13010004

**Published:** 2024-12-24

**Authors:** Sandra Gagulic, Ana Bártolo, Alda Marques

**Affiliations:** 1 Research Center on Physical Activity, Health and Leisure (CIAFEL), Faculty of Sport, University of Porto, 4200-465 Porto, Portugal; sandra.gagulic@ipiaget.pt; 2 Insight: Piaget Research Center for Ecological Human Development, Instituto Piaget-ESS/Piaget, 3515-776 Viseu, Portugal; 3 CINTESIS@RISE, CINTESIS.UPT, Portucalense University, 4200-072 Porto, Portugal; ana.bartolo@upt.pt; 4 Respiratory Research and Rehabilitation Laboratory (Lab3R), School of Health Sciences (ESSUA) and Institute of Biomedicine (iBiMED), University of Aveiro, Agras do Crasto—Campus Universitário de Santiago, Building 30, 3810-193 Aveiro, Portugal

**Keywords:** pediatrics, adherence, physical activity, endurance, quality of life

## Abstract

**Background/Objectives:** Exercise for children with cystic fibrosis leads to well-known health benefits. However, maintaining regular activity is challenging due to the daily demands of academics, clinical care, and family tasks. Home-based exercise programs offer a more adaptable alternative, fitting into family schedules. This study evaluated the effectiveness of the “KidMove” program, a parent-supervised, tailored, home exercise regimen. **Methods:** A quasi-experimental study was conducted with an intervention group (IG) and a wait-list control group (CG). The “KidMove” program lasted 12 weeks and included 35 exercises targeting endurance, resistance, flexibility, and neuromotor training. The primary outcome, endurance, was measured with the Modified Shuttle Walking Test, while secondary outcomes included body composition, resistance, flexibility, postural control, respiratory function, and health-related quality of life. Data were collected at baseline and post-intervention. A per-protocol analysis was conducted with generalized estimating equations (GEEs). **Results:** Forty-six children aged 10 ± 4 years (6 to 18 years), mostly male (n = 24; 52.2%), participated. Significant improvements were observed in the Modified Shuttle Walking Test [Wald χ^2^ = 14.24, *p* < 0.001], postural control [Wald χ^2^ = 3.89, *p* = 0.048], knee flexibility [Wald χ^2^ = 5.58, *p* = 0.018], and emotional functioning [Wald χ^2^ = 9.34, *p* = 0.002] categories. **Conclusions:** The “KidMove” program offers a practical, family friendly alternative to center-based exercise by empowering parents to support their children’s physical activity at home, endurance, flexibility, and emotional well-being, while reducing the logistical challenges.

## 1. Introduction

Cystic fibrosis is a complex, multi-systemic disorder primarily characterized by pulmonary complications that significantly impact morbidity and mortality rates [1,2]. It is characterized by progressive chronic airway obstruction and infections since infancy and childhood [3], as well as lung function decline in adolescence [4], which lead to a reduced exercise capacity, muscle resistance, balance, shoulder and chest mobility, bone density, and an increased risk of fractures [5]. These complications contribute to limitations in daily activities, a loss of independence, and a decline in overall quality of life [1,6].

Exercise programs are recognized as vital non-pharmacological interventions for individuals with cystic fibrosis, ideally beginning in early childhood. They help mitigate cystic fibrosis-related morbidity by improving physical health, fitness levels, and social engagement [7], although minor effects on lung function and health-related quality of life have also been observed [5]. Maintaining adequate physical fitness is associated with a favorable disease prognoses and reduced hospitalizations [5,8,9].

However, children with CF often face physical challenges, such as reduce pulmonary function, muscle weakness, and fatigue, which can create significant social barriers, including feelings of inadequacy and withdrawal from group activities. These challenges are further amplified by the absence of tailored exercise opportunities in schools and community programs [5]. Tailored exercise programs that are specifically designed to address individual needs and preferences have been shown to be more effective than generic approaches in improving both physical and psychological outcomes, highlighting the importance of personalized strategies in supporting this population [9,10,11]. These programs can be designed to address specific postural control issues, incorporating balance, resistance, and flexibility training that are personalized for the individual [5,7]. Nevertheless, most treatments for cystic fibrosis are complex and require a significant amount of time to administer. Even when children receive the most suitable treatment and appropriate guidance, adherence to treatment recommendations in children with cystic fibrosis is reported to be less than 50% [12]. A recent meta-analysis has found that support from parents and their modeling behaviors were related to children’s physical activity levels [13]. Therefore, balancing the benefits and burden of exercise is crucial not just for individuals, but also for families and health professionals, ensuring adherence to exercise as a standard therapeutic approach [11,14]. Community- or home-based exercise programs have been suggested as promising alternatives since they can be more flexibly administered and minimize the risk of cross-infection [15]. Nevertheless, their effects on physical fitness, lung function, infection status, antibiotic usage, health-related quality of life, adherence, and satisfaction remain limited and inconsistently documented [5]. A recent systematic review reported the effectiveness of home-based rehabilitation on pulmonary function, functional capacity, and health-related quality of life in children and adolescents (6 to 20 years old) with cystic fibrosis, emphasizing that home-based rehabilitation may be an alternative when conventional center-based programs are not feasible [14]. Exercise interventions, especially those tailored to individual needs, have been proposed as crucial for managing these complications, yet adherence remains a major issue, especially in children. Given the potential of home-based exercise programs to provide a more flexible and feasible alternative to traditional center-based approaches, this study aimed to assess the effectiveness of a tailored home-based exercise program, “KidMove”, on children with cystic fibrosis with parents’ supervision. We hypothesized that a home-based exercise program will improve endurance (primary outcome) and other health-related physical fitness components (body composition, muscle resistance, flexibility, and neuromotor outcomes), as well as lung function and health-related quality of life in children with cystic fibrosis.

## 2. Materials and Methods

### 2.1. Study Design

A quasi-experimental study with two arms (intervention group vs. wait-list control group) was conducted to assess the effects of a home-based exercise program: “KidMove”. Ethical approval was obtained from the Ethics Committee of the Centro Hospitalar de Coimbra and the Ethics Committee Centro Hospitalar do Porto (CHUC-004-13), where recruitment took place. Informed consent was obtained from the parents or guardians of each participant prior to any data collection, and privacy was ensured in accordance with the European regulation (EU 2016/679). All procedures were conducted in accordance with the ethical standards of the National Research Committee, the 1964 Helsinki Declaration, and its subsequent amendments or comparable ethical standards. The study was unblinded, as participants were aware of the interventions. This study adheres to the Consolidated Standards of Reporting Trials (CONSORT) statement for non-randomized studies [16] and Template for Intervention Description and Replication (TIDiER) [17] guidelines (see Supplementary Materials).

### 2.2. Participants and Procedure

Participants were eligible for inclusion in the study if they were 18 years of age or younger, had a respiratory function test result of FEV1 > 40%, and regularly attended their routine hospital appointments. Individuals were excluded if they were mechanically ventilated, had a musculoskeletal condition that could interfere with their physical assessment, or experienced a recent (within the last month) acute respiratory exacerbation [3].

Recruitment was conducted face to face using a convenience sampling method. Children and adolescents were initially identified by their attending pediatricians, who provided a brief explanation of the study to the whole family. Those who met the eligibility criteria and expressed an interest in participating were subsequently contacted by a member of the research team (SG), who provided detailed information about the study and addressed any remaining questions.

A statistical power analysis was conducted using G*Power 3 (University of Düsseldorf, Germany) to determine the required number of participants for the study. This calculation was based on data from previous studies that evaluated the impact of a home-based exercise program on the Modified Shuttle Walking Yest (MSWT) [18]. The sample size analysis indicated that 13 participants per group would be needed to detect a large effect size on the MSWT (m) with 80% power and a 5% significance level. To account for this, a target of 26 participants per group was set for the study [19]. Additionally, Whitehead and colleagues recommend that for a study aimed at achieving 90% power with a two-sided 5% significance level and considering medium standardized effect sizes, the pilot sample sizes should include a minimum of 15 participants per arm [20]. This guideline further supports the rationale behind our sample size decision, ensuring that our study is adequately powered to detect meaningful effects.

### 2.3. Intervention

Participants in the intervention group completed a personalized home-based exercise program, “KidMove”, alongside standard treatment, which included antibiotics, bronchodilators, pancreatic enzyme supplements, and airway clearance techniques.

The intervention began with a face-to-face baseline assessment, during which researchers provided individualized guidance on appropriate exercise intensities and instructed participants on monitoring key physiological parameters, including heart rate (targeting 70–80% of the maximum heart rate) and perceived exertion using the modified Borg scale (target range: 4–6). Participants were also educated on recognizing warning signs and symptoms, emphasizing the importance of stopping exercise immediately if their heart rate exceeded the recommended maximum or if they experienced extreme coughing or a dyspnea perceived exertion rating of 8–10 on the modified Borg scale [16].

Caregivers were actively involved in the intervention. An initial training session was delivered, where caregivers were instructed on their role in supporting and supervising the exercise program. This included strategies for encouraging and motivating participants, as well as participating in some exercises themselves to foster engagement. Caregivers were also trained to monitor and document key details of the intervention, such as the type, duration, and frequency of physical activities, using a structured activity diary. These diaries were periodically reviewed by the research team to ensure adherence to the exercise program and to address any challenges or concerns raised by participants or caregivers. By integrating caregiver involvement and structured monitoring, the “KidMove” program sought to create a supportive environment, reinforcing adherence to the intervention while maintaining a focus on participant safety.

The program lasted 12 weeks, with the recommended frequency of exercise 3 to 5 times per week, and consisted of a total of 35 exercises targeting endurance, resistance, balance, and flexibility training, supervised by parents. Exercises were selected based on each participant’s preferences and strategies to enhance adherence were based on previous studies, such as the Fitness Challenge Program (FitKitTM) [17]. Participants were instructed to choose exercises from each category according to their preference and that they should progress if the exercises were too easy (<4) in perceived exertion in the modified Borg scale. Physical activity levels were documented using self-reported diaries, detailing exercise type, duration, supervision status, and associated symptoms [21]. Before the intervention, education on hydration and breathing techniques during exercise was implemented by one member of the research team (SG), aiming to enhance awareness and facilitate sustained engagement in physical activities.

The exercise regimen was carefully designed to provide a comprehensive and structured program, incorporating a warm-up phase (5–10 min) comprising range-of-motion exercises, stretching, low-intensity aerobic exercises, and breathing techniques. Endurance training sessions lasting 20–30 min, with activities such as swimming, running, cycling, skipping, aerobic classes, step aerobics, and trampolining. The intensity of endurance training was individually prescribed using the MSWT [22], targeting a perceived exertion of 4–6 on the modified Borg scale [23]. Exercises were tailored to match participants’ fitness levels and preferences, ensuring both engagement and safety. Resistance training sessions (15 min) started with one set of 10 repetitions and progressed to six sets of 10–15 repetitions per set, with a 60 s rest between sets. Resistance exercises included bodyweight activities, such as push-ups, squats, lunges, and the use of free weights (e.g., a packet of rice or bottles). Balance and flexibility training (5 min each) included static and dynamic exercises arranged at increasing difficulty levels [24], such as tandem walking and one-leg stands. Flexibility training also lasted 5 min and focused on major muscle groups. These exercises included yoga-inspired stretches and static holds. The cool-down period (10 min) mirrored activities from the warm-up phase [25,26]. The intensity of all exercises was monitored and adjusted according to the participant’s perceived fatigue and dyspnea, targeting a range of 4–6 on the modified Borg scale.

Participants and parents were instructed to accumulate 150 min of exercise per week, aligning with the recommendations from the American College of Sports Medicine [26], and report the type of exercise and adverse events in the activity diary. To improve program adherence, parents and teenagers received biweekly mobile messaging and weekly phone calls from researchers. Adverse events during exercise were reported in the activity diary. A structured guide was followed during telephone calls consisting of gathering data on encountered challenges and adjustments needed in the exercise. Participants were advised to promptly consult their attending physician or researcher regarding concerns about exacerbations, signs of respiratory distress, monitoring, or exercise-related issues.

### 2.4. Outcome Measures

Data were collected at baseline and after 12 weeks by the same assessor (SG; physiotherapist) who was experienced in administering the selected outcome measures.

The primary outcome was the endurance component, assessed using the MSWT. The MSWT involved participants walking or running on a 10 m course with cones, guided by auditory cues that progressively increase speeds from 0.5 m/s, escalating by 0.17 m/s per minute until exhaustion or an inability to complete the exercise within the specified time frame [18,27,28]. Participants completed the MSWT twice with a minimum mandatory 30 min rest period between tests (until returning to their baseline vital signs) to mitigate learning effects. The best performance was kept for analysis. Key metrics recorded included total distance covered, maximum speed achieved, number of shuttle runs completed, as well as reasons for test cessation to ensure safety and an accurate interpretation of results. This test is well known for its dependability in young people with cystic fibrosis [29]. It also shows good test–retest reliability (r = 0.99, *p* < 0.01), sensitivity to change in aerobic fitness, and a substantial correlation with VO_2_max in cystic fibrosis. Physiological responses, including heart rate and peripheral oxygen saturation, were monitored using a pulse oximeter (Moretti, FS10) before and after each MSWT. Fatigue and dyspnea levels were assessed using the modified Borg scale, which closely correlates (0.6–0.8) with VO_2_peak [30]. The minimal clinically important difference of the MSWT has been found to be 97 m [29].

The remaining health-related physical fitness components were included as secondary outcomes and were assessed with body mass index (BMI) (weight/height^2^) for body composition (SECA^®^ mechanical scale and WHO AnthroPlus software, version 2007); handheld dynamometer (microFET2, Hoggan Health, The Best Salt Lake City, UT, USA) in kilogram-force (KgF) for muscle strength [31]; the modified sit and reach test (MSRT) [32] for flexibility; and the star excursion balance test (SEBT) [33] for the neuromotor component. Additionally, a spirometry (Spirobank II USB R©, MIR, Rome, Italy) was conducted, according to the American Thoracic Society/European Respiratory Society guidelines [34], to assess lung function and the values obtained for the forced expiratory volume in one second (FEV_1_), and the forced vital capacity in liters and percentage predicted were registered (FCV). Finally, the cystic fibrosis questionnaire—revised (CFQ-R) was used to assess health-related quality of life [35].

Sociodemographic and clinical data were collected to characterize the sample, as well as recruitment and retention indicators through (1) participation rate: participants volunteering to participate/eligible participants × 100; and (2) study dropout (post-test): participants who did not complete the post-test/enrolled participants × 100. Adverse events during exercise were reported in the activity diary.

### 2.5. Data Analysis

Descriptive statistics were used to describe the sample and to evaluate retention and effectiveness indicators. Absolute and relative frequencies, measures of central tendency, and/or dispersion were utilized. To evaluate the intervention’s effectiveness (examining both between-group and within-group effects for each outcome), data were analyzed using methods appropriate for correlated panel data, specifically generalized estimating equations (GEE) modeling. The quasi-likelihood information criterion (QIC) was used to determine the optimal correlation structure and best-fitting model within the GEE analysis. For all outcomes, a Gamma distribution was chosen, as it yielded the lowest QIC. Statistical analyses were conducted using IBM SPSS Statistics for Windows, version 28.

## 3. Results

### 3.1. Characterization of Study Participants at Baseline

**Figure 1:**
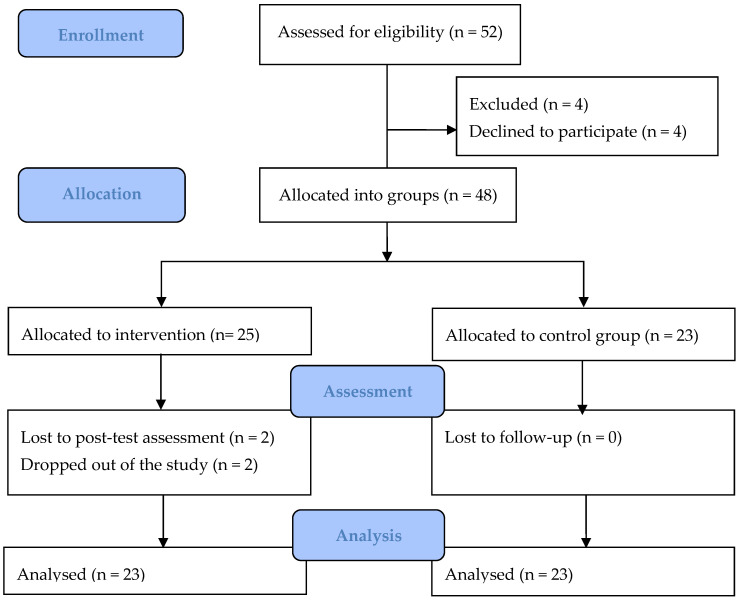
Consolidated standards of reporting trials (CONSORT) diagram of the included participants with cystic fibrosis in the home-based exercise program (“KidMove”).

Fifty-two children and/or adolescents with cystic fibrosis were eligible and were referred to participate in the study, 48 of whom agreed to take part. Two participants allocated to the intervention group dropped out of the study and did not complete the intervention. Based on a per-protocol approach, 46 participants were included in the final analysis (23 participants in each group; see Figure 1). The participation rate in the study was 92.3% and the dropout rate was 4.3%. No adverse events were reported.

Participants were 10 ± 4 years old (6 to 18 years old), mostly male (n = 24; 52.2%), and were attending preschool and primary school (n = 25; 54.3%). Approximately 54.3% (n = 25) did not have physical education classes in a school context. At the time of recruitment, 13% (n = 6) of the children had experienced an exacerbation, requiring hospitalization, within the past 3 months. Detailed characteristics of the participants for each group are presented in Table 1. No differences were found between groups at baseline.

### 3.2. Effectiveness of the Intervention

Following a per-protocol approach, a significant group-by-time interaction effect was observed, with the intervention leading to improved performance on the MSWT, highlighted the positive effects of the “KidMove” program on the endurance of participants (Table 2). In the intervention group, the increase in distance achieved during the MSWT was 99.56 m, indicating a real and clinically meaningful improvement, whereas no significant improvement was observed in the control group. The data are presented as mean ± standard deviation (SD), along with frequency and percentage.

A significant increase in scores from pre-test to post-test was observed in the intervention group, while in the control group, these scores remained similar (although with a slight improvement) from baseline to post-test. Regarding the quality of life, an interaction effect was identified in the dimension in the intervention group, while no significant changes were noted in the control group (Table 2).

## 4. Discussion

The present study showed that the tailored home-based exercise program, “KidMove”, is safe and effective in improving the endurance of children with cystic fibrosis.

Improvements of secondary outcomes, including the neuromotor component (excursion distance and anterior right stance), muscle resistance (knee flexion and wrist extensor resistance), and health–related quality of life (emotional functioning), highlighted the potential of this approach.

Overall, the findings underscore the benefits of structured exercise programs for children with cystic fibrosis. Participants demonstrated enhancements in endurance, muscle strength, balance, and flexibility, contributing to improved functional and emotional health. These results align with the existing literature, as exercise interventions have been linked to improvements in endurance and affective responses associated with the disease, particularly in pediatric contexts [6,10,18,36,37,38]. 

Home-based exercise programs overcome barriers related to the daily routine overload of families, leading to greater adherence and promising results in improving endurance, resistance, and overall physical functioning. The “KidMove” program demonstrated consistency with previous evidence, as the retention rate was 95.7%, and a group-by-time interaction effect was observed for the primary outcome, with the intervention group showing a greater change in MSWT from baseline to post-intervention [10,18].

In addition to this statistical difference, the minimal clinically important difference (mswt) ≥ 97.08 m [29] was reached. These findings suggest that the proposed intervention confers advantages to children and adolescents with cystic fibrosis, producing outcomes similar to those attained by center-based methods [28,39]. Moreover, the “KidMove” program outperformed other home-based approaches which have demonstrated smaller changes in endurance, without evidence of changes in minimal clinical difference [40]. This difference may be attributed to the greater flexibility of the intervention proposed in this study, which allowed participants to select exercises based on their preferences, thereby enhancing adherence and increasing engagement in the exercises.

It is also important to highlight that “KidMove” distinguishes itself by integrating a multimodal intervention option, which includes flexibility, balance/neuromotor skills, and endurance exercises. This may further explain its positive outcomes in secondary indicators, such as postural control and knee flexion. Flexibility exercises have not been a major focus in most interventions [38], yet they may also positively impact the circulatory system’s postural control and balance ability [41].

The positive impact of structured exercise programs on health-related quality of life across multiple domains—including physical, emotional, social, and academic performance—has been well-documented in children with cystic fibrosis [35]. The tailored training program provided substantial benefits, such as enhanced endurance, muscle resistance, balance, flexibility, and postural control, which collectively improved functional mobility and reduced fall risks. Additionally, flexibility exercises contributed to better joint mobility and circulation, while emotional well-being improvements alleviated psychological burdens and fostered adherence to the program [42]. This study replicated the findings at the level of health-related quality-of-life emotional functioning, demonstrating improvements in the intervention group and a decline in the control group.

This is particularly relevant, given that cystic fibrosis has been recognized as a disease with a significant emotional impact on children and their families, further underscoring the broad applicability of this intervention approach.

Despite these results, some limitations are worth noting. Although the study included a control group, its quasi-experimental design limits the ability to establish a direct causal relationship between the “KidMove” program and the observed outcomes. The lack of randomization, a key feature of randomized controlled trials, may introduce selection bias, affecting the comparability between groups. Additionally, the sample size may have been insufficient to detect effects in some secondary outcomes, limiting the generalizability of the results. Although the program’s flexibility, which permits participants to select their exercises daily, can be seen as a strength, it also introduces a methodological bias that may result in varied outcomes, complicating the standardization of the intervention. Additionally, the lack of a long-term follow-up hinders the assessment of the program’s sustainability and its enduring impacts on both primary and secondary outcomes.

Future studies should consider adopting more rigorous designs, such as randomized controlled trials, along with long-term follow-ups to assess the sustainability of the intervention’s benefits. Including qualitative data from participants and their families could also provide valuable insights into the intervention process, helping refine and optimize the program for better outcomes.

## 5. Conclusions

This study demonstrated that the personalized home-based exercise program, “KidMove”, is both safe and effective at enhancing the endurance of children with cystic fibrosis. Moreover, the study also supports the idea that combining different types of exercises, such as endurance, resistance, and neuromotor control, can significantly improve physical and emotional health in young cystic fibrosis individuals. The intervention enhanced emotional health, particularly in the CFQ-R Emotion domain, suggesting a link between physical activity and mental well-being in patients with chronic illnesses, like cystic fibrosis. This study emphasizes home-based personalized exercise programs as a safe and effective alternative to the one-size-fits-all approaches to improve health outcomes in cystic fibrosis patients.

## Figures and Tables

**Table 1:** Baseline characteristics of participants with cystic fibrosis included in the study (n = 46).

Variable	Total Sample	Intervention Group (IG; n = 23)	Control Group (CG; n = 23)	t-Value/χ^2^	*p*
	n (%)	n (%)	n (%)		
Age, years (M(SD))	10.37 (3.86)	10.3 (4.09)	10.3 (3.70)	0.03	0.970
Sex, n (%)					
Male	24 (52.2)	11 (47.8)	13 (56.5)		
Female	22 (47.8)	12 (52.2)	10 (43.5)	3.0	0.223
Education				0.18	0.980
Elementary school	25 (54.3)	12 (52.2)	13 (56.5)		
5th–6th grade	8 (17.4)	4 (17.4)	4 (17.4)		
7th–9th grade	6 (13.0)	3 (13.0)	3 (13.0)		
Secondary school	7 (15.2)	4 (17.4)	3 (13.0)		
Physical exercise at school (yes)	21 (45.7)	13 (56.5)	8 (34.8)	2.19	0.139

**Table 2:** Results at baseline and 12 weeks after the tailored home-based exercise program in children with cystic fibrosis with parents’ supervision, “KidMove”, according to the per-protocol analyses (n = 46).

Outcome	Outcome Measure	Intervention Group (IG) (n = 23)		Control Group (CG) (n = 23)		Group-Time Effect		
		T0	T1	T0	T1	χ^2^	*p* *	B (95% CI) †
		M (SE)	M (SE)	M (SE)	M (SE)			
BMI, Kg/m^2^		17.4 (0.68)	17.1 (0.6)	17.6 (0.5)	17.5 (0.52)	2.2	0.131	0.261 (−0.078, 0.600)
	FEV1, % predicted	78.8 (2.5)	79.2 (2.4)	85.8 (2.3)	85.6 (2.3)	1.7	0.184	−0.578 (−1.431, 0.274)
	FVC, % predicted	77.0 (2.1)	78.2(1.9)	85.4 (1.7)	85.1 (1.8)	3.1	0.076	−1.583 (−3.33; 0.165)
	Modified Shuttle Walking Test (m)	515.2 (24.8)	614.7 (26.4)	495.2 (18.9)	530.4 (20.3)	**14.2**	**<0.001**	**−64.35** **(−97,775; −30.92)**
Neuromotor component	Star excursion balance test—posteromedial right	79.9 (1.1)	85.2 (1.0)	72.69 (2.2)	77.6 (1.7)	0.12	0.720	−0.378 (−2.449, 1.692)
	Star excursion balance test—medial	65.2 (2.0)	71.0 (2.1)	38.8 (2.2)	45.3 (2.2)	0.24	0.623	0.630 (−1.880, 3.141)
	Star excursion balance test—anterior right	70.4 (1.6)	75.6 (1.6)	69.4 (2.7)	73.2 (2.7)	**3.8**	**0.048**	**−1.383 (−2.756, −** **0.009)**
Muscle Strength	HHDMVS-HHD, Kg/F							
	HFMVS-HHD Kg/F)	17.5 (1.1)	18.9 (1.2)	17.1 (1.0)	18.4 (1.0)	0.25	0.613	−0.130 (−0.636, 0.375)
	HABMVS-HHD Kg/F	15.6 (1.3)	16.9 (1.4)	15.7 (1.3)	16.9 (1.2)	0.07	0.788	−0.061 (−0.504, 0.382)
	KEMVS-HHD Kg/F	19.5 (1.7)	21.3 (1.8)	19.4 (1.5)	20.8 (1.6)	2.7	0.097	−0.474 (−1.034, 0.087)
	KFMVS-HHD Kg/F	18.3 (1.4)	19.7 (1.5)	17.0 (1.0)	17.8 (1.0)	**5.5**	**0.018**	**−** **0.617 (−1.130, −** **0.105)**
	ADFMVS-HHD Kg/F	13.9 (0.9)	15.4 (1.0)	13.9 (0.6)	15.2 (0.6)	0.48	0.487	-0.187 (0.2688, −0.714)
	SFMVS-HHD Kg/F	11.5 (0.9)	12.4 (1.0)	11.9 (0.8)	12.7 (0.8)	0.48	0.487	−0.135 (−0.515, 0.245)
	SABMVS-HHD Kg/F	11.7 (1.1)	12.2 (1.2)	11.9 (1.1)	12.3 (1.1)	1.8	0.180	−0.170 (−0.417, 0.078)
	EFMVS-HHD Kg/F	13.7 (1.1)	14.8 (1.1)	14.2 (0.8)	15.0 (0.8)	2.7	0.095	−0.270 (−0.586, 0.047)
	EEMVS-HHD Kg/F	10.3 (1.0)	10.8 (0.9)	11.8 (0.9)	12.5 (0.9)	2.5	0.108	0.274 (−0.060, 0.608)
	WEMVS-HHD Kg/F	9.2 (0.91)	9.7 (0.95)	9.4 (0.6)	9.8 (0.6)	**4.0**	**0.044**	**−** **0.187 (−.369, −** **0.005)**
Flexibility (m)	Modified Sit Reach Test	15.7 (0.93)	18.5 (1.0)	14.2 (0.7)	16.6 (0.8)	0.60	0.437	−0.387 (−1.362, 0.588)
HRQoL	CFQ-R Physical	71.2 (3.1)	74.6 (3.2)	63.7 (3.2)	66.7 (3.3)	0.01	0.904	−0.387 (−6.664, 5.890)
	CFQ-R Emotion	71.7 (3.2)	77.3 (2.8)	84.4 (2.1)	83.6 (2.8)	**9.3**	**0.002**	**−6.435** **(−10.562, −2.308)**
	CFQ-R Eating	52.6 (4.2)	54.1 (3.8)	57.0 (3.0)	57.5 (2.9)	0.10	0.741	−0.756 (−5.246, 3.734)
	CFQ-R Treatment Burden	66.2 (3.3)	70.5 (2.7)	73.9 (2.5)	74.9 (2.3)	2.2	0.130	−3.378 (−7.754, 0.997)
	CFQ-R Social	54.9 (3.4)	58.4 (3.0)	58.6 (2.3)	61.3 (1.6)	0.20	0.654	−0.837 (−4.497, 2.822)
	CFQ-R Body image	72.7 (4.3)	78.3 (3.3)	69.6 (2.5)	73.9 (2.3)	0.25	0.612	−1.207 (−5.863, 3.450)
	CFQ-R Respiratory	73.5 (1.9)	82.8 (1.3)	76.2 (2.1)	80.9 (2.2)	3.0	0.079	−4.600 (−9.737, 0.537)
	CFQ-R Digest	74.8 (4.3)	82.6 (3.1)	89.8 (3.1)	91.3 (3.0)	2.1	0.144	−6.287 (−14.726, 2.152)

Abbreviations: BMI: body mass index; FEV: forced expiratory volume; FVC: forced vital capacity; MSWT: Modified Shuttle Walking test; HF-HHD: hip flexor maximum voluntary isometry strength—hand held dynamometry; HAB-HHD: hip abductor maximum voluntary isometry strength—hand held dynamometry; KE-HHD: knee extensor maximum voluntary isometry strength—hand held dynamometry; KF-HHD: knee flexor maximum voluntary isometry strength—hand held dynamometry; ADF-HHD: ankle dorsiflexor maximum voluntary isometry strength—hand held dynamometry; SF-HHD: shoulder flexor maximum voluntary isometry strength—hand held dynamometry; SAB-HHD: shoulder abductor maximum voluntary isometry strength—hand held dynamometry; EF-HHD: elbow flexor maximum voluntary isometry strength—hand held dynamometry; EE, HHD: elbow extensor maximum voluntary isometry strength—hand held dynamometry: HAD-HHD: hip adductor maximum voluntary isometry strength—hand held dynamometry; HE-HHD: hip extensor maximum voluntary isometry strength—hand held dynamometry; WE-HHD: wrist extensor maximum voluntary isometry strength—hand held dynamometry; MSRT: modified sit and reach; Cystic Fibrosis Questionnaire—Revised. * *p*-values for type-III GEE model effects tested using the Wald Chi-Square test; values in bold represent the statistically significant differences at *p* < 0.05; † unstandardized coefficient and 95% confidence interval reflect the group–time effect corresponding to group 1 * time 1 (upper and lower values, respectively).

## Data Availability

The data presented in this study are available on request from the corresponding authors due to confidentiality agreements.

## Supplementary Materials

The following supporting information can be downloaded at: https://www.mdpi.com/article/10.3390/healthcare13010004/s1, Table S1: TIDieR (Template for Intervention Description and Replication) checklist.
